# Mark Hallett and the renaissance of functional neurological disorder^☆^

**DOI:** 10.1016/j.prdoa.2026.100464

**Published:** 2026-06-16

**Authors:** Jon Stone

**Affiliations:** Centre for Clinical Brain Sciences, University of Edinburgh, Edinburgh, EH16 4SA, UK

**Keywords:** Functional neurological disorder, Mark Hallett, Psychogenic, Conversion disorder, Pathophysiology, Volition

## Abstract

This article traces the contributions of Mark Hallett (1943–2025) to Functional Neurological Disorder (FND) from 2003 to his death. Mark brought his expertise in motor physiology, free will, volition and agency to this neglected but common condition. He raised awareness of our need to understand its pathophysiology, natural history and treatment, and then became a key researcher investigating abnormalities of brain networks found in individuals with the condition, as well as its multifactorial aetiology. He played a pivotal role in organising international meetings and books on functional movement disorder and FND, culminating in his role as the first president of the International FND Society (2019–2022). In 2024, Mark Hallett gave the 2024 American Academy of Neurology Wartenberg lecture, recalling “la lésion dynamique” proposed by Charcot as the pathophysiology of FND in the 19th century. This lecture was emblematic of the renaissance of interest in FND after a long period of neglect and his huge contribution to our understanding of the disorder.

Since Mark Hallett died in November 2025, he has had more ‘in memoriam’ articles, named conferences and special article collections published in his honour than any neurologist I can remember in my career. In this article I'm glad to revisit Mark's contribution to functional neurological disorder (FND) and especially functional movement disorder.

I met Mark for the first time relatively late in his career, but early in the renaissance of FND. He led the organisation of a meeting co-organised by Stanley Fahn, Joe Jankovic, Anthony Lang, Bob Cloninger and Stuart Yudofsky in Atlanta in 2003. At the time, I had just completed my own PhD in functional limb weakness. I was somewhat astonished but thrilled that so many luminaries of movement disorders were congregating to discuss the topic of functional movement disorder when the amount of literature at that time was modest. That meeting later turned into a 2006 book, with chapters on many aspects of the topic, including pathophysiology, treatment and history, that had been previously neglected [1].

This conference and book were emblematic of Mark Hallett's contribution to FND. Huge in scope, highly collaborative, with a focus on trying to understand the basics of mechanism, but also with an eye to the global extent of human misery associated with this unique disorder. In this article, I will discuss Mark's contribution to the field under the headings of phenomenology, conceptual advances related to agency and volition linking to his work in motor physiology, pathophysiology including functional neuroimaging and genetics, and global field-building.

Mark's path through FND has been an important driver of the field's renaissance and tells its own story of where FND was in the past and where it may be going in the future. I will use the term ‘functional’ instead of ‘psychogenic’ or other terms used in older papers for consistency, unless relevant.

## 1 Phenomenology – from “conversion disorder” to ‘rule in’ diagnosis

In the 1990s, a series of seminal articles describing the features of psychogenic movement disorder as it was known then, appeared in the literature. Although there had always been sporadic papers on conversion disorder, or hysteria, these series of papers were the first to properly apply the rigour of movement disorder phenomenology to the patients seen in the clinic (Table 1). The key point in all of them was that functional movement disorders could be described in their own terms. They had their own set of clinical characteristics, just like restless legs syndrome, or Parkinson's disease, even if they didn't yet have a pathophysiology that neurologists might understand. For example, functional tremor could be recognised by variable frequency or entrainment, functional myoclonus by sudden onset and improvement with distraction and functional dystonia by a clenched fist or an inverted ankle.

**Table 1:** Key early case series of phenomenology in functional movement disorder.

Year	Authors and Title	N	Clinical Observations
**1988**	Fahn S, Williams DT. **Psychogenic dystonia.**[4]	21	Sudden onset, fixed posture, remission. Suggested division into documented (with remission), clinically established, probable and possible
**1989**	Koller W, Lang AE, Vetere-Overfield B, Findley L, Cleeves L, Factor S, Singer C, Weiner W. **Psychogenic tremors.**[5]	24	Abrupt onset, distractibility, changing amplitude and frequency, ability to perform selective tasks despite severe tremor.
1991	Lempert T, Brandt T, Dieterich M, Huppert D, How to identify psychogenic disorders of stance and gait, [6]	37	Momentary fluctuations of stance and gait; excessive slowness or hesitation; Romberg test unsteadiness improved by distraction; uneconomic postures; "walking on ice" gait pattern, sudden buckling of the knees, usually without falls.
**1993**	Monday K, Jankovic J. **Psychogenic myoclonus.**[6]	18	Changing distribution, amplitude and frequency, Improvement with distraction, placebo or hypnosis.
**1995**	Lang AE, Koller WC, Fahn S. **Psychogenic parkinsonism.**[7]	14	Tremor entrainment, rigidity improving with distraction, arm cradled, lack of decrement in slowness. Normal Fluorodopa 18 scans.

This recognition that FND has a consistent neurological phenomenology was not new. It followed a long tradition going back to the 19th century, starting with Todd, who first described the ‘dragging gait’ or functional paralysis, through Reynolds, Charcot, Janet and many others [2]. In 1922, the London neurologist Henry Head felt confident enough about the stereotypical features of FND to write in the BMJ that its clinical signs were “*as definite and specific as those of any other disease. Hysteria is sometimes said to ‘imitate’ organic affections; but this is a highly misleading statement. The mimicry can only deceive an observer ignorant of the signs of hysteria or content with a perfunctory examination*” [3].

The history of FND is remarkable for its non-linearity, and rediscovery of lost knowledge. FND had been dominated in the 20th century by a psychiatric model, and conversion disorder especially. This meant that by the publication of DSM-IV in 1984 the problem was known as “Conversion Disorder” and required the presence of a stressful event, but only the absence of an explanatory neurological disease.

The ‘new’ phenomenology papers were in direct contradiction to a diagnosis of exclusion, since they had recognised the typical features possessed by the patients, which were largely those of their movements, and not the psychological make-up of the person who had them. Indeed, one could argue that ‘rule-in’ diagnosis and recognition that a neurologist was needed to do that, has been a cornerstone, alongside studies of the mechanism of FND in the brain itself, of the reinvigoration of FND within the speciality of neurology.

Mark wasn't part of these early phenomenological papers which appeared slowly over a 15-year period before the galvanising effect of the 2003 Atlanta meeting. Later, his group at the National Institutes of Health (NIH) in Bethesda demonstrated using accelerometry that it was possible to distinguish functional from essential and Parkinsonian tremor [8]. There had been case reports and mentions of using contralateral limb tapping to entrain functional tremor [5], [9], [10], but this study was the first to systematically show, in six individuals, how useful this clinical sign was, and how much it could separate out a functional tremor group. It was especially useful to show that a 3 Hz tap was more discriminatory than a 4 Hz or 5 Hz tap. Tremor entrainment remains one of the most helpful clinical signs in functional movement disorder, although in practice, the transient cessation of the tremor, rather than the variation of tremor frequency that was captured by this study, is most helpful at the bedside.

Arguably, his later contributions to phenomenology were more about gathering together these descriptions, in books and review articles [11], [12], [13], [14], [15], some from meetings and projects discussed further below.

One particular clinical sign, however, anticipatory limb jerking even when a tendon hammer stops short of contact, was one he was fond of showing and writing about [16]. I was glad to be able to name this the ‘Hallett sign’, with colleagues, in an article after his death. [17].

## 2 Volition, free will, agency and FND

The issue of how symptoms in FND, such as paralysis, tremor or seizures, relate to volition, free will and a human being's sense of agency has always been a central one for clinicians meeting patients with FND.

As far back as 1837, nearly two hundred years ago, Benjamin Brodie had observed “*In hysterical paralysis, it is not that the muscles are incapable of obeying the act of volition, but that the function of volition is not exercised*” [18]. In 1873, Paget more pithily observed, ‘*They say “I cannot”; it looks like “I will not”, but it is “I cannot will”* ‘ [19] strongly acknowledging the influence of Brodie. It's possible to pick up strands of the same observation infrequently across the 20th century [20], but efforts to frame FND as, at least at one level, a disorder of the machinery of volition or agency were generally swamped by those focusing on a psychological and especially emotional/psychological trauma level of understanding.

Mark Hallett came to free will through his interest in movement disorders and especially the Bereitschaftspotential, which offered the opportunity for a biomarker of voluntary movement [21]. In his 2007 article ‘Volitional control of movement: the physiology of free will’ [22] he describes functional movement disorders as one of the ‘neurological disorders of volition’ along with tics, alien hand, and passivity phenomena in schizophrenia. He explored and expanded the classic Libet experiment [23] which found that the time of conscious intention to act appeared to be later than the Bereitschaftspotential, indicating that the brain had already decided to move. One of his achievements in movement disorders was to develop the neuroscience and philosophical problem of free will in a way that was clinically applicable to those studying movement disorders.

His interest in FND, therefore, which is evident only in his published work from 2003 onwards [8], was inspired not only by an appreciation of clinical need, but also by a fascination and overlap with his interests in motor control and agency.

I heard Mark speak on free will on several occasions and was struck each time by the clarity and logic that he brought to the topic. His conclusion about what the Libet experiment is telling us, at least as I remember it, was especially memorable to me. “Yes, your brain appears to decide before you're aware of the decision, but your brain is still ‘you’ “ [22]. I find myself thinking of that frequently when I talk to people who punch themselves during their functional seizures, or when trying to explain how we might help someone gain control over movements they have no agency over. The individuals do not experience actions as voluntary, but at some level, it is still their brain that is responsible, and with a disconnection that can potentially be retrieved.

Studies had already shown that movement preparation, the conscious intention to move, and agency (the feeling that it is *you* that has made the movement) were likely separable processes. His work in functional movement disorder provided a surprisingly direct parallel to the work that had gone before on voluntary movement in healthy controls using experimental manipulation of agency and volition. Some of these parallels are shown in Table 2.

**Table 2:** Experimental studies on healthy controls in agency and volition have informed subsequent work in patients with functional movement disorder.

	Healthy Control Experimental Studies	Functional Movement Disorder
1. Libet clock experiments	Intentional binding can be disrupted using transcranial direct current stimulation over the pre-supplementary motor area [24]	FMD patients have reduced gap between conscious intention to move (W) and actual movement (M) compared to controls [25], [26] Decreased action-effect binding (self-agency task) [27]
2. Agency and Right temporoparietal junction (TPJ)	Reduced Right TPJ activity correlates with decreased agency in a joystick task [28], [29]	Reduced Right TPJ activity in functional tremor vs patient's own voluntary tremor. [30], [31]
3. Bereitschaftspotential	Present before voluntary movement [21]	Helped demonstrate 1) some forms of functional movement disorder – especially propriospinal myoclonus are commonly related to FMD. [32] 2) The machinery of voluntary movement is intact despite experience of being involuntary.

## 3 Brain networks and *La Lésion Dynamique*

**Fig. 1:**
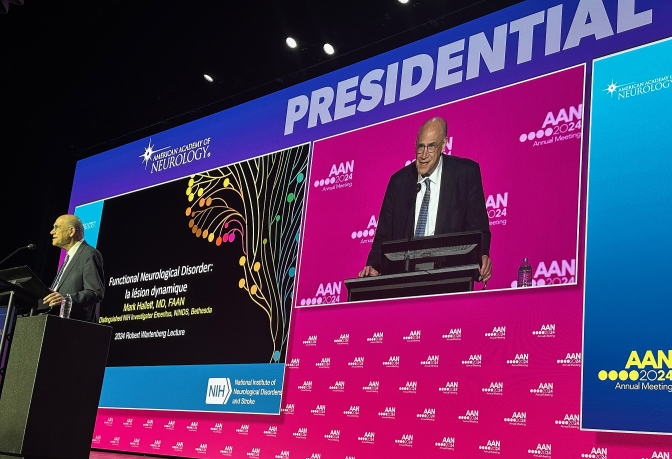
Mark Hallett presenting the Wartenberg lecture at the 2024 American Academy of Neurology – photo courtesy of Joseph Jankovic.

In 2024, Mark Hallett gave the Wartenberg lecture at the American Academy of Neurology annual meeting on the topic of FND [33] (see Fig. 1). His framing recalled Charcot's conclusion regarding the pathophysiology of a flaccid left arm paralysis after an injury in an 18-year-old man called ‘Pin’ [34]. Charcot eventually concluded that– “*We have here unquestionably one of those lesions which escape our present means of anatomical investigation, and which, for want of a better term, we designate dynamic or functional lesions*”.

The lecture, along with other co-authored studies [14], [35], [36], have explored “la lésion dynamique” in FND now that we *do* have a means of anatomical investigation. Several fMRI studies of patients with functional movement disorder [37], including his own [38], have found hyperactivity or changes in connectivity between the amygdala and limbic areas and motor areas, especially the supplementary motor area. Developmental or antecedent changes in brain structures related to adverse experience, potentially under conditions of excessive cortisol, have also been highlighted in his work [39], [40].

An fMRI study of individuals with functional tremor who were asked to make a voluntary tremor, led by Valerie Voon, is still, to my mind, one of the most persuasive functional imaging studies of FND [30]. It showed hypoactivation of the right temporoparietal junction, an important node in the network of sense of agency. More recent functional imaging studies have explored how machine learning might predict FND compared to controls [41], how interoceptive processing may be abnormal in FND [42], [43] and how network connectivity analyses may predict outcomes in FND [44].

**Fig. 2:**
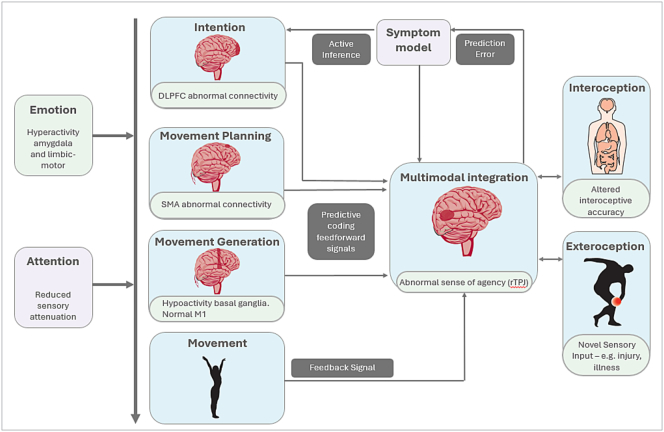
Neural mechanisms of FND (redrawn from Lancet Neurology review on FND- Hallett et al. [14]) DLPFC – Dorsolateral prefrontal cortex. SMA- Supplementary Motor Area. TPJ – Temporoparietal junction.

Fig. 2 is redrawn from our joint Lancet Neurology review, which gave both a clinical and mechanistic/aetiological view of the disorder [14]. It places data from FND functional imaging and neurophysiological studies in a predictive coding framework which Mark Edwards and colleagues first applied to the field in 2012 [45] and is well rehearsed elsewhere.

Primavera Spagnolo collaborated with Mark to pioneer the study of genetic vulnerability to FND [46], [47] in small case-control studies with fewer than 70 individuals. These have suggested tryptophan hydroxylase 2 (TPH2) gene polymorphisms and DNA methylation characteristic of chronic stress and pain as possible candidates. We have started to see much larger genome-wide studies in FND. For example, a study in 10,910 individuals with functional seizures found no genome-wide significant loci and SNP heritability of only 2% [48]. Studying gene-brain interaction effects or gene-phenotype modifiers may likely be a more productive research route.

## 4 Havana syndrome and post-infectious myalgic encephalomyelitis/chronic fatigue syndrome

In the last few years of his time at NIH, Mark contributed to related research on two contested syndromes. NIH carried out a reassessment of the controversial “Havana syndrome”, mostly US diplomatic staff posted in Cuba who developed a range of symptoms including sensory sensitivity, headache, fatigue and cognitive symptoms [49]. My understanding is that his second author position on this paper reflected the fact that Mark saw most of these patients himself as part of extensive week-long testing. Initial studies of these patients published in JAMA [50], [51] suggested they had evidence of brain damage, perhaps consistent with some kind of remote weapon. The final NIH conclusion was that 24% had functional neurological disorder, mostly persistent postural perceptual dizziness and a range of other sometimes non-specific symptoms, but no evidence of brain damage or unusual findings on structural or functional neuroimaging.

In a similarly thorough deep phenotyping study of individuals with post-infectious myalgic encephalomyelitis/chronic fatigue syndrome, 17 individuals with the condition underwent an enormous battery of tests including functional neuroimaging, immune, autonomic, cognitive, cardiopulmonary, cerebrospinal fluid and muscle strength testing [52]. One of the key findings was a motor physiology one. Compared to healthy volunteers, there was a reduction in ability to maintain a moderate grip force, which correlated, as in FND, with decreased activity of the right temporoparietal junction. There was an increase in sympathetic but a decrease in parasympathetic activity, which could not be attributed to anxiety, as well as downregulations of cerebrospinal fluid tryptophan metabolites and some interesting immunological leads, although no consistent pattern of immune dysregulation.

## 5 Global field building and the FND society

Arguably, Mark's greatest legacy in FND is in the energy he provided towards global field building. His career was marked by the founding of so many previous international societies that when Mark, Alan Carson and I came to negotiate the administration fees to start the FND Society in 2019, he managed to obtain us a “frequent flyer” discount with the management company.

Mark Hallett did not organise the first FND-themed meeting in the modern era. This was a 1999 Oxford meeting organised by Peter Halligan, Christopher Bass and John Marshall, which led to a multidisciplinary book [53]. There had also been US-based volumes on functional seizures in the past led by John Gates starting with a symposium in 1990 [54]. As ever, the history of most topics like this is rarely black and white. Research and clinical interest in FND and its historical forbears didn't cease completely over the 20th century but certainly was at a low level compared to what came subsequently [55].

However, the 2003 Atlanta meeting on “Psychogenic Movement Disorders”, which Mark had a key role in leading, was the first truly international meeting. It later produced the first multi-author book specifically on functional movement disorder in 2006 [1]. The three-day meeting translated the Movement Disorder Society ethos of late-night video sessions, focus on phenomenology and pathophysiology, but added in psychiatric, psychological, historical and evolutionary contributions. It provided an exciting sense of possibility, and of a global network of researchers engaged in the problem from the multiple perspectives needed to properly understand it.

A subsequent meeting in 2009 in Washington covered ‘Psychogenic and other conversion disorder’ with a wider remit and led to a second multi-author book in 2011 [56], co-edited with Valerie Voon and similar editors to the first volume. It had a large collection of videos published in CD format with it, which is well worth digging out if you can find it and is in the process of being republished.

By the time Mark was ready for another international meeting, he had, together with Alan Carson and me, completed co-editing the *Handbook of Neurology* volume on FND [13]. Most older neurologists, at least, will know this series that has been running since 1968 covering every aspect of neurology. It was a signal of how hidden FND had been from the curriculum that there had never been a volume on it over that time. Volume 139, however, edited by Mark, myself and Alan Carson, eventually appeared in 2016 with 51 chapters and 662 pages [13]. It was the first attempt at a truly comprehensive overview of all aspects of FND, including chapters on dizziness, cognition, hearing, urinary retention and other neglected areas. This time round, the conference was organised as a way of expanding on the book. The meeting, which Mark decided should be called the “3^rd^ International Conference on Functional (Psychogenic) Neurological Disorders”, was held in Edinburgh in September 2017. Alan had hired a much larger 600-seater hall, we advertised widely, and the meeting was a great success. Somewhat to my astonishment, we managed to fill the venue and raise enough money, together with Mark's previous FND meeting funds, sponsorship from the Movement Disorder Society, National Institutes of Health, American Epilepsy Society and others to start the FND society in 2019.

Mark took on the role as first president of the FND Society until our fourth (covid delayed) meeting in Boston in 2022. Over that time, the society grew steadily and now has over 1400 multidisciplinary members (summer 2026). I must admit I watched on with some confusion as Mark developed detailed bylaws and set up numerous committees when we were only a fledgling organisation. It has taken time for me to see the positive effect his administrative talent had on the growth of the society and its global multidisciplinary network of clinicians and researchers. Unusually, those who have FND or just have an interest can also join as community members and attend meetings. When “FND Portal”, a pseudonym for an online FND patient advocate, gave a lecture at our 2022 meeting, he received a standing ovation, which for me represented a landmark in a disorder where so often the patient's voice has been missing. FND Portal's essay ‘Cadenza for a fractured consciousness’ is highly recommended and, in my view, the best modern piece of writing on FND not (yet) to be found in a journal [57].

## 6 Attitudes to FND in neurology – a crisis highlighted but not yet resolved

Mark Hallett's published work on FND had an important role in changing attitudes within neurology. As a senior and respected figure in Neurology, working at the heart of US national neurological research, people listened when he wrote in 2006 that functional movement disorder was a ‘crisis for neurology’. This paper opened with this sentence “*The nature of the crisis is that there are many patients, we don't understand the pathophysiology, we often don't know how to make the diagnosis, we don't know how to treat the patients, the patients don't want to hear that they have a psychiatric disorder and they go from doctor to doctor, psychiatrists don't seem interested anyway, and the prognosis is terrible.”*

It was ironic that Mark Hallett, working at a national referral centre at the NIH, should have had so much exposure to one of the commonest clinical conditions in neurological practice. One of the reasons for this was the ‘undiagnosed diseases network’ where patients with undiagnosed or rare conditions could be seen at NIH if their case was sufficiently puzzling. Knowledge about FND was so scarce, and diagnostic reluctance so great, that Mark found that 30% of his quaternary movement disorder clinic patients had this diagnosis [16]. To many, this would have been an annoyance. To Mark, this was a clinical opportunity to help and the rationale for a research program. One of them was the patient advocate FND Portal mentioned earlier. I suspect that stream of FND patients is now less. Certainly, I recall patients with FND often being presented in UK neurology ‘grand rounds’ when I was training in the late 1990s because they were a “diagnostic mystery”, when in reality they had little described or undescribed variants of FND unfamiliar to those seeing them.

When Mark revisited his ‘crisis for neurology’ paper in 2019, his new title was ‘*Functional movement disorders: is the crisis resolved?’*. He concluded that it was not, but that “progress is strong”, and he hoped that in another 10 years the “crisis will be over” when people with FND are “managed well with multidisciplinary teams”. That change is certainly happening, but three more years is not long to achieve that aim. FND is now part of the neurology training curriculum in Europe [58], there are American Academy Guidelines for the management of functional seizures [59], and the European Academy of Neurology ones are on the way for FND. However, on the ground, people with FND still find it hard to find multidisciplinary care, training remains very limited for many health professionals in this area, and in many regions of the world, not much has changed in FND practice over time.

## 7 The FND renaissance and Mark Hallett

It is accurate to describe the last 20 years or so of activity around FND as a renaissance. Similar levels of interest and multidisciplinary enquiry occurred in the late 19th and early 20th century, but then the field fell into relative obscurity. There would, I think, still have been an FND renaissance without Mark Hallett. But it would not have been charged with the same energy and global reach that occurred with him. We would perhaps just be getting round to forming the FND society now in 2026 had he not helped us to do so in 2019. He brought respect to the field for neurologists but also translational ideas from his work on voluntary movement and free will, neurophysiology, functional imaging, genetics, and synthesis of brain network discoveries which continue to exert a strong influence on the field. His mentorship of hundreds of clinicians, by extension to the members and board of the FND Society as its first president, and to me personally, leave both an immediate and lasting legacy.

The sum total of Mark Hallett's contribution is being recognised by the FND Society with the creation of the Hallett Award. This is the first award the society has created. It recognises those who have contributed to the work of the society and FND research and care internationally. It is fitting that the first two awardees were involved in all those early functional movement disorder papers and conferences, Anthony Lang and the co-editor of this special issue, Joseph Jankovic.

## Financial disclosure/conflict of interest concerning the manuscript

None.

## CRediT authorship contribution statement

**Jon Stone:** Writing – review & editing, Writing – original draft, Conceptualization.

## Funding sources

None.

## Declaration of competing interest

Professor Stone reports honoraria from UptoDate, personal fees from Expert Witness Work, grants from National Research Scotland, outside the submitted work; He runs a self help website, www.neurosymptoms.org, for patients with Functional Neurological Disorder. He is President of the FND Society from 2026-2028.
